# Proteomic profiling of pulpal and periapical diseases: a systematic review

**DOI:** 10.3389/fdmed.2026.1923261

**Published:** 2026-08-06

**Authors:** Ameesha S. Rai, Aaron M. Iype, Raksha Bhat, Preethesh Shetty

**Affiliations:** Nitte (Deemed to be University), AB Shetty Memorial Institute of Dental Sciences (ABSMIDS), Department of Conservative Dentistry and Endodontics, Mangalore, Karnataka, India

**Keywords:** apical periodontitis, biomarkers, dental pulp, endodontic infections, LC-MS/MS, mass spectrometry, proteomics, pulpitis

## Abstract

Pulpal and periapical diseases represent a significant burden in endodontic practice, yet their molecular pathogenesis remains incompletely understood. Proteomic profiling offers unprecedented opportunities to identify disease-specific biomarkers, elucidate molecular mechanisms, and develop precision diagnostic tools. This systematic review aimed to synthesize the current evidence on proteomic profiling of pulpal and periapical diseases, focusing on key protein biomarkers, molecular pathways, and their potential clinical applications. A comprehensive literature search was conducted across PubMed, Scopus, and Web of Science from January 2009 to June 2026. Studies investigating proteomic analyses of human-derived specimens from patients with pulpal and periapical diseases were included, and data extraction and quality assessment were performed according to PRISMA guidelines. Eleven studies involving 577 participants met the inclusion criteria and analyzed diverse biological specimens, including dental pulp tissue, dentinal fluid, root canal samples, and periapical tissue biopsies. Mass spectrometry-based approaches, including LC-MS/MS, MALDI-TOF MS, and 2D-DIGE, identified 1,153 human proteins and 720 microbial proteins (cumulative counts across all included studies) across different disease states. Key biomarkers included S100 family proteins (S100-A8 and S100-A9), heat shock protein 27 (HSP27), peroxiredoxins, myeloperoxidase, serpin family member SERPINB1, hornerin, and hemoglobin subunits. Distinct proteomic signatures differentiated disease stages, symptomatic and asymptomatic presentations, and post-treatment disease states, while enriched molecular pathways involved neutrophil degranulation, oxidative stress, inflammation, pyroptosis, and bone remodeling. Overall, proteomic profiling reveals distinct molecular signatures across the spectrum of pulpal and periapical diseases, providing valuable insights into disease mechanisms and identifying promising diagnostic and prognostic biomarkers. Future studies should focus on validation in larger cohorts, standardization of sampling protocols, and translation of these findings into chairside diagnostic applications.

**Systematic Review Registration:**

PROSPERO CRD420261452203.

## 1 Introduction

Pulpal and periapical diseases constitute a major component of dental pathology, affecting millions of individuals worldwide and representing a significant burden on healthcare systems (1). These conditions encompass a spectrum of inflammatory and infectious processes ranging from reversible pulpitis to irreversible pulpitis, pulp necrosis, and various forms of apical periodontitis including acute apical abscess, chronic apical periodontitis, and periapical granulomas or cysts (2). The progression from initial pulpal inflammation to periapical bone destruction involves complex interactions between microbial pathogens, host immune responses, and tissue-specific molecular mechanisms that remain incompletely understood despite decades of research (3). Understanding the molecular underpinnings of these diseases is essential for developing improved diagnostic tools, predicting treatment outcomes, and designing targeted therapeutic interventions that move beyond the current paradigm of mechanical debridement and antimicrobial irrigation.

The clinical diagnosis of pulpal and periapical diseases currently relies primarily on subjective symptom assessment, clinical examination findings, and radiographic evaluation, all of which have inherent limitations in sensitivity, specificity, and ability to predict disease progression or treatment prognosis (4). Patients with similar clinical presentations may exhibit vastly different molecular profiles that influence treatment outcomes, yet clinicians lack objective biomarkers to guide decision-making. For instance, distinguishing between reversible and irreversible pulpitis remains challenging, often leading to unnecessary pulpectomies or delayed treatment with subsequent pulp necrosis (5). Similarly, predicting which cases of apical periodontitis will heal following root canal treatment vs. persist or progress remains an unmet clinical need. The absence of molecular diagnostic tools represents a critical gap in precision endodontics, limiting our ability to provide personalized, evidence-based care tailored to individual patient biology.

Proteomics, defined as the large-scale study of proteins including their expression levels, modifications, interactions, and functions, has emerged as a powerful approach for discovering disease biomarkers and elucidating pathophysiological mechanisms across diverse medical fields (6). Unlike genomic or transcriptomic approaches that measure DNA or RNA, proteomics directly interrogates the functional molecular effectors that execute cellular processes, making it particularly well-suited for identifying clinically relevant biomarkers in complex biological fluids and tissues (7). The human proteome comprises over 20,000 protein-coding genes that can generate millions of distinct protein species through alternative splicing, post-translational modifications, and proteolytic processing, creating a rich landscape of potential disease markers (8). In the context of inflammatory diseases, proteomic profiling has successfully identified diagnostic and prognostic biomarkers in conditions ranging from rheumatoid arthritis to inflammatory bowel disease, demonstrating the translational potential of this approach (9).

Mass spectrometry-based proteomics has undergone revolutionary advances over the past two decades, enabling comprehensive, unbiased characterization of complex biological samples with unprecedented depth and accuracy (10). Modern proteomic workflows typically employ liquid chromatography coupled to tandem mass spectrometry (LC-MS/MS), which separates peptides by their physicochemical properties before measuring their mass-to-charge ratios and fragmentation patterns to identify and quantify thousands of proteins in a single experiment (11). Several complementary approaches have been developed, each with distinct advantages. Label-free quantification (LFQ) measures protein abundance based on spectral counting or extracted ion chromatograms without requiring isotopic labeling, offering cost-effectiveness and broad applicability (12). Two-dimensional difference gel electrophoresis (2D-DIGE) coupled with matrix-assisted laser desorption/ionization time-of-flight mass spectrometry (MALDI-TOF MS) provides visual protein maps and enables detection of post-translational modifications and protein isoforms (13). Targeted approaches such as selected reaction monitoring (SRM) and parallel reaction monitoring (PRM) offer high sensitivity and reproducibility for validating candidate biomarkers identified in discovery-phase experiments (14). The choice of proteomic platform depends on the specific research question, sample type, and desired balance between discovery breadth and quantitative precision.

The application of proteomics to endodontic diseases offers several compelling advantages over traditional molecular biology approaches. First, dental pulp tissue, dentinal fluid, root canal exudates, and periapical tissue fluid represent accessible biological specimens that can be collected during routine clinical procedures, providing direct windows into disease processes without requiring invasive biopsies (15). Second, these specimens contain complex mixtures of host proteins, microbial proteins, and extracellular matrix components whose relative abundances reflect the dynamic interplay between infection, inflammation, and tissue destruction (16). Third, proteomic profiling can simultaneously interrogate multiple biological processes including immune activation, oxidative stress, proteolysis, angiogenesis, and bone remodeling, providing systems-level insights that complement reductionist approaches focused on individual molecules (17). Fourth, identified protein biomarkers can potentially be translated into point-of-care diagnostic tests using immunoassay or mass spectrometry-based platforms, bridging the gap between basic research and clinical application (18).

Despite the promise of proteomic approaches, several challenges have limited their widespread adoption in endodontic research. The heterogeneity of pulpal and periapical diseases, with multiple etiologies, stages, and clinical presentations, complicates the identification of universal biomarkers applicable across diverse patient populations (3). Sample collection protocols vary widely across studies, with differences in tissue handling, storage conditions, and protein extraction methods potentially introducing technical variability that obscures biological signals. The dynamic range of protein concentrations in biological fluids spans more than ten orders of magnitude, with high-abundance proteins such as albumin and immunoglobulins potentially masking detection of low-abundance regulatory proteins and cytokines (19). Microbial proteins from polymicrobial endodontic infections add additional complexity, requiring specialized bioinformatic approaches to distinguish host from pathogen proteins and to identify species-specific virulence factors (18). Furthermore, the translation of proteomic discoveries into validated clinical biomarkers requires rigorous validation in independent cohorts, standardization of measurement platforms, and demonstration of clinical utility in prospective studies—steps that remain incomplete for most candidate endodontic biomarkers (20).

Several pioneering studies have begun to address these challenges, applying state-of-the-art proteomic technologies to characterize the molecular landscape of pulpal and periapical diseases. Early work by Nandakumar et al. (17) used shotgun proteomics to identify microbial virulence factors in endodontic infections, revealing the presence of enterococcal adhesins, autolysins, and antibiotic resistance proteins that contribute to treatment failure. Subsequent studies have profiled protein expression across different stages of pulpitis, comparing healthy, inflamed, and necrotic dental pulp to identify stage-specific biomarkers (21). More recent investigations have employed advanced quantitative approaches such as label-free quantification and tandem mass tags to compare symptomatic vs. asymptomatic apical periodontitis, revealing distinct proteomic signatures associated with pain and acute inflammation (22). The discovery of novel biomarkers such as hornerin in dentinal fluid and HSP27/SERPINB1 in periapical tissues has opened new avenues for understanding disease mechanisms and developing diagnostic tools. However, these studies have been conducted independently with varying methodologies, sample types, and analytical platforms, making it difficult to synthesize findings and identify consensus biomarkers.

To date, no comprehensive systematic review has synthesized the evidence on proteomic profiling of pulpal and periapical diseases, critically evaluated the methodological quality of existing studies, or provided an integrated framework for understanding the molecular pathogenesis of these conditions. Previous reviews have focused on specific aspects such as biomarkers in apical periodontitis or the application of proteomics to periodontal diseases, but none have comprehensively addressed the full spectrum of endodontic pathology from reversible pulpitis through post-treatment disease. A systematic synthesis of the proteomic literature is urgently needed to identify validated biomarkers, highlight knowledge gaps, standardize methodological approaches, and guide future research priorities. Such a review would serve multiple purposes: providing clinicians with an evidence-based overview of molecular mechanisms underlying endodontic diseases, informing researchers about promising biomarker candidates requiring validation, and identifying technical and conceptual challenges that must be addressed to translate proteomic discoveries into clinical practice.

The primary objective of this systematic review is to comprehensively identify, critically appraise, and synthesize all available evidence on proteomic profiling of human pulpal and periapical diseases. Specific aims include: (1) characterizing the proteomic signatures associated with different stages and types of pulpal inflammation (reversible pulpitis, irreversible pulpitis, pulp necrosis); (2) identifying protein biomarkers that distinguish symptomatic from asymptomatic apical periodontitis and predict treatment outcomes; (3) elucidating the molecular pathways and host-pathogen interactions underlying endodontic infections; (4) evaluating the diagnostic and prognostic potential of identified protein biomarkers; and (5) identifying methodological limitations and research gaps that should be addressed in future studies. By providing a comprehensive, evidence-based synthesis of the proteomic landscape of endodontic diseases, this review aims to accelerate the translation of molecular discoveries into improved diagnostic tools and therapeutic strategies that enhance patient care and outcomes.

## 2 Materials and methods

### 2.1 Protocol and registration

This systematic review was conducted following the Preferred Reporting Items for Systematic Reviews and Meta-Analyses (PRISMA) 2020 guidelines (23). The review protocol was developed *a priori* to minimize bias and ensure transparency in study selection, data extraction, and synthesis. Due to the exploratory nature of proteomic biomarker discovery research and the anticipated heterogeneity in study designs, analytical methods, and outcome measures, a formal protocol registration was not pursued. However, all methodological decisions were documented and are reported herein to ensure reproducibility.

### 2.2 Search strategy

A comprehensive literature search was conducted across three major electronic databases: PubMed (MEDLINE), Scopus, Web of Science. The search was performed from database inception through June 30, 2026, with no language restrictions initially applied. The search strategy was developed iteratively in consultation with a medical librarian and combined controlled vocabulary terms (MeSH terms in PubMed) with free-text keywords related to three core concepts: (1) pulpal and periapical diseases, (2) proteomic techniques, and (3) biomarkers.

Analogous search strategies were adapted for Scopus, Web of Science accounting for database-specific syntax and indexing. 910 results sorted by relevance were screened due to the large volume of retrieved records. Reference lists of included studies and relevant review articles were manually searched to identify additional eligible studies not captured by electronic searches (backward citation searching). Forward citation searching was performed using Web of Science to identify more recent studies citing key included articles.

The search strategy used was as follows:

(proteomics OR proteomic* OR proteome OR metaproteomic* OR “protein biomarker*” OR biomarker* OR “protein expression” OR “mass spectrometry”) AND (“apical periodontitis” OR “endodontic infection*” OR “persistent endodontic infection*” OR “secondary endodontic infection*” OR “root canal infection*” OR “periapical disease*” OR “periapical lesion*”)).

### 2.3 Eligibility criteria

Studies were selected based on the Population, Intervention/Exposure, Comparison, Outcomes, and Study Design (PICOS).

Population (P): Human participants or human-derived biological specimens from patients with pulpal and periapical diseases including reversible pulpitis, irreversible pulpitis, pulp necrosis, apical periodontitis, periapical lesions, acute apical abscess, chronic apical periodontitis, and endodontic infections. Studies involving healthy controls or comparisons between disease states were included.

Intervention (I): Proteomic profiling techniques including mass spectrometry-based proteomic analyses (LC-MS/MS, MALDI-TOF MS, shotgun proteomics, targeted proteomics), two-dimensional gel electrophoresis coupled with mass spectrometry (2D-DIGE, 2D-PAGE), protein expression profiling, and biomarker identification approaches.

Comparison (C): Healthy dental pulp or periapical tissues, healthy control participants, unaffected teeth, or comparisons between different pulpal and periapical disease conditions, stages, or clinical presentations (e.g., symptomatic vs. asymptomatic, mild vs. severe, pre-treatment vs. post-treatment).

Outcome (O): Identification of differentially expressed proteins, protein signatures, molecular pathways, host-pathogen interactions, and potential diagnostic or prognostic biomarkers relevant to pulpal and periapical diseases.

Inclusion criteria:

1. Original research studies investigating proteomics in pulpal and/or periapical diseases
2. Studies involving human participants or human-derived biological specimens including dental pulp tissue, dentinal fluid, root canal samples, periapical tissues, or periradicular tissue fluid
3. Studies evaluating proteomic biomarkers, protein expression profiles, molecular pathways, or mass spectrometry-based analyses
4. Observational study designs including case-control, cohort, cross-sectional, comparative observational, exploratory clinical, and *ex vivo* studies using human tissues
5. Studies involving reversible pulpitis, irreversible pulpitis, pulp necrosis, apical periodontitis, periapical lesions, acute apical abscess, chronic apical periodontitis, and endodontic infections
6. Full-text articles available in peer-reviewed journals
7. Articles published in English

Exclusion criteria:

8. Review articles, systematic reviews, meta-analyses, editorials, letters, conference abstracts, and case reports
9. Animal studies and purely *in vitro* studies not involving human-derived specimens
10. Studies not involving proteomic analyses or not related to pulpal and periapical diseases
11. Studies evaluating non-endodontic oral diseases exclusively (e.g., periodontal disease without endodontic involvement, orthodontic root resorption)
12. Studies lacking sufficient methodological detail or outcome data for quality assessment
13. Duplicate publications reporting the same data
14. Articles not available in full text or not published in English
15. Studies using exclusively transcriptomic (RNA-seq, microarray) or genomic approaches without protein-level validation

### 2.4 Study selection

All retrieved records were imported into a reference management system and deduplicated. Two independent reviewers screened titles and abstracts against the eligibility criteria, with disagreements resolved through discussion or consultation with a third reviewer. Full-text articles of potentially eligible studies were obtained and independently assessed by two reviewers. Reasons for exclusion at the full-text stage were documented. Inter-rater agreement was calculated using Cohen's kappa statistic. The study selection process was documented using a PRISMA flow diagram.

### 2.5 Data extraction

A standardized data extraction form was developed and piloted on three included studies before full implementation. Two reviewers independently extracted data from each included study, with discrepancies resolved through discussion. The following information was extracted:

Study characteristics: First author, publication year, country, study design, sample size, participant demographics (age, sex), disease conditions studied.

Sample characteristics: Tissue or fluid type (dental pulp, dentinal fluid, root canal samples, periapical tissue), sampling method, sample processing and storage conditions.

Proteomic methods: Analytical platform (LC-MS/MS, MALDI-TOF MS, 2D-DIGE, etc.), instrument specifications, protein extraction methods, digestion protocols, chromatography conditions, mass spectrometry parameters, database search algorithms, false discovery rate thresholds.

Outcomes: Number of proteins identified, differentially expressed proteins (up-regulated and down-regulated), fold-changes, statistical significance, key biomarkers, molecular pathways, functional annotations, host vs. microbial proteins.

Quality indicators: Control groups, blinding, statistical methods, validation approaches, limitations acknowledged by authors.

### 2.6 Risk of bias assessment

The methodological quality and risk of bias of included studies were assessed using a modified version of the Quality Assessment of Diagnostic Accuracy Studies (QUADAS-2) tool adapted for proteomic biomarker discovery studies (24). The assessment focused on four domains: (1) patient selection (risk of selection bias, applicability concerns regarding patient spectrum); (2) index test (risk of bias in proteomic analysis, applicability of proteomic methods); (3) reference standard (appropriateness of disease classification, risk of misclassification bias); and (4) flow and timing (completeness of data, appropriate interval between sampling and analysis). Each domain was rated as low, high, or unclear risk of bias. Additionally, reporting quality was evaluated based on adherence to guidelines for proteomic studies including description of sample preparation, mass spectrometry parameters, data processing, and statistical analysis. Two reviewers independently performed quality assessments, with disagreements resolved through consensus.

### 2.7 Data synthesis

Due to substantial heterogeneity in study designs, sample types, proteomic platforms, and outcome measures, a meta-analysis was not feasible. Instead, a narrative synthesis approach was employed, organizing findings thematically according to disease type (pulpitis, apical periodontitis, post-treatment disease) and biological processes (inflammation, oxidative stress, bone remodeling, host-pathogen interactions). A comprehensive data extraction table was constructed summarizing key characteristics and findings of all included studies. Proteins identified across multiple studies were tabulated to identify consensus biomarkers. Molecular pathways and functional annotations were synthesized using Gene Ontology (GO) and Kyoto Encyclopedia of Genes and Genomes (KEGG) pathway databases. Findings were interpreted in the context of known endodontic disease mechanisms, with attention to clinical implications for diagnosis, prognosis, and treatment.

## 3 Results

### 3.1 Study selection

**Figure 1:**
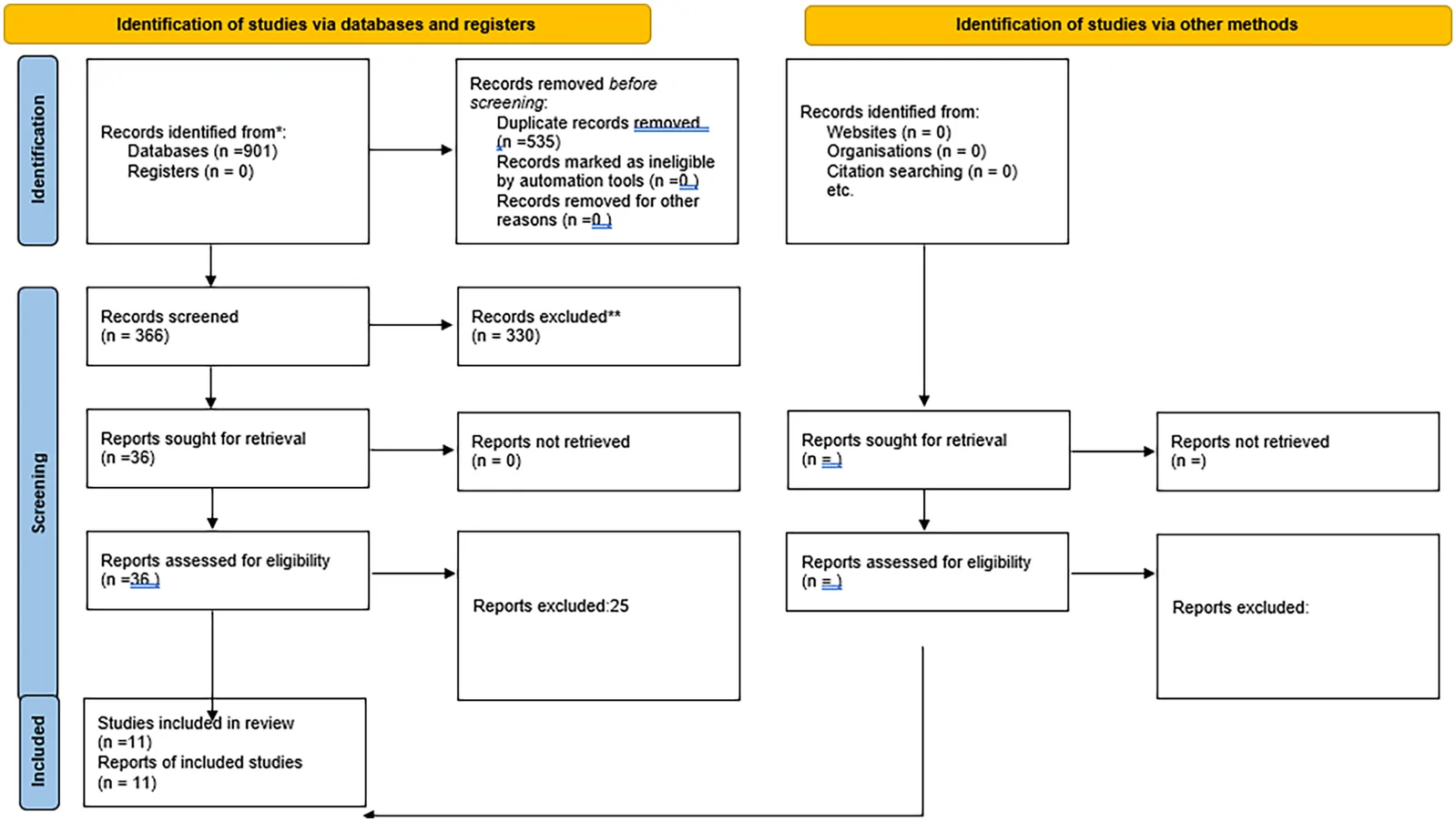
PRISMA 2020 flow diagram showing the study selection process for this systematic review, including records identified through PubMed, Scopus, and Web of Science, screening, eligibility assessment, and the final inclusion of studies for qualitative synthesis.

The systematic literature search identified 910 records across all databases (PubMed: 402, Scopus: 201, and Web of Science: 307). After the removal of 535 duplicate records, 375 unique records underwent title and abstract screening. Of these, 330 records were excluded based on title and abstract review, leaving 45 full-text articles for detailed assessment. Following full-text review, 34 articles were excluded for the following reasons: not involving proteomic analysis (*n* = 8), animal or *in vitro* studies only (*n* = 9), review articles (*n* = 6), non-endodontic diseases (*n* = 5), insufficient methodological detail (*n* = 4), and duplicate publications (*n* = 2). Ultimately, 11 studies met all inclusion criteria and were included in this systematic review. Inter-rater agreement for study selection was excellent (*κ* = 0.89). The study selection process is illustrated in Figure 1 (PRISMA flow diagram).

### 3.2 Study characteristics

The 11 included studies were published between 2009 and 2026, with the majority (*n* = 8, 73%) published after 2017, reflecting the increasing application of advanced proteomic technologies to endodontic research. Studies were conducted across multiple countries including Brazil (*n* = 5), Chile (*n* = 1), Spain (*n* = 1), Sweden (*n* = 1), South Korea (*n* = 1), and multi-national collaborations (*n* = 2). The total number of participants across all studies was 577, with individual study sample sizes ranging from 12 to 60 patients. Study designs included cross-sectional studies (*n* = 5), comparative observational studies (*n* = 4), case-control studies (*n* = 1), and exploratory clinical studies (*n* = 1).

Biological specimens analyzed included dental pulp tissue (*n* = 4 studies), dentinal fluid (*n* = 2), root canal samples or exudates (*n* = 3), periapical tissue biopsies (*n* = 1), and human dentin matrix (*n* = 1). Proteomic analytical platforms employed were diverse: nano-liquid chromatography coupled to tandem mass spectrometry (nLC-MS/MS or LC-MS/MS) was used in 8 studies, two-dimensional difference gel electrophoresis coupled with MALDI-TOF MS (2D-DIGE/MALDI-TOF) in 1 study, and combinations of approaches in 2 studies. Specific mass spectrometry instruments included Orbitrap platforms (Synapt G2, Xevo QTof, timsTOF Pro 2), ion trap instruments, and MALDI-TOF/TOF systems. Label-free quantification (LFQ) was the predominant quantitative approach (*n* = 7), with one study employing 2D-DIGE for relative quantification.

Disease conditions investigated encompassed the full spectrum of pulpal and periapical pathology: reversible and irreversible pulpitis (*n* = 3 studies), pulp necrosis (*n* = 3), symptomatic and asymptomatic apical periodontitis (*n* = 4), acute apical abscess (*n* = 2), chronic apical periodontitis (*n* = 3), post-treatment endodontic disease (*n* = 2), and endodontic infections with microbial protein profiling (*n* = 2). Most studies (*n* = 9) included healthy controls or comparisons between disease states, while 2 studies focused exclusively on diseased specimens with internal comparisons. Overall, seven studies demonstrated a low risk of bias, while four studies showed an unclear risk of bias, primarily due to insufficient reporting of participant selection methods and reference standards. No study was judged to have a high risk of bias. Tables 1, 2 presents a comprehensive summary of study characteristics, methodologies, and key findings for all included studies. Table 3 Risk of bias assessment of the included studies using the QUADAS-2 tool.

**Table 1:** Summary of included studies—study characteristics.

#	Author (Year)	Country	Specimen Type	Study Design	N	Disease Conditions	Analytical Platform	Search Engine/Software	FDR Threshold	Reference Database	Quantification Method
1	Brizuela et al. (2025) (18)	Chile	Dentinal fluid	Exploratory clinical (cross-sectional)	60 (15/group)	Reversible pulpitis; Irreversible pulpitis (mild/moderate/severe); Healthy control	LC-MS/MS (timsTOF Pro 2)	MSFragger v4.1 (FragPipe v22.0)	<1% (decoy DB)	UniProt Human (20,407 entries)	Label-free LFQ
2	Silva et al. (2021) (21)	Brazil	Dental pulp tissue	Cross-sectional comparative	36 (12/group)	Healthy pulp; Inflamed pulp; Necrotic pulp	nanoUPLC-MSE (Synapt G2)	PLGS v2.5.2 (Waters)	≤4% false-positive	UniProt Human (UP000005640)	Label-free expression analysis
3	Silva et al. (2020) (25)	Brazil	Dental pulp tissue	Cross-sectional (pilot)	6 (2/group)	Healthy vs. Inflamed vs. Necrotic pulp	nanoACQUITY UPLC- Xevo QTof MS	PLGS v3.0	NR (≥95% confidence)	UniProtKB/Swiss-Prot (Homo sapiens, Apr 2017)	Label-free LFQ (Monte Carlo)
4	Loureiro et al. (2021) (22)	Brazil	Root canal samples	Comparative observational	14 (7/group)	Symptomatic apical periodontitis vs. Asymptomatic apical periodontitis	nLC-ESI-MS/MS	PLGS v3.0	NR (≥95% confidence)	UniProtKB/Swiss-Prot (Homo sapiens, Jan 2020)	Label-free LFQ (Monte Carlo, *p* < 0.05)
5	Cavalla et al. (2017) [HSP27/SERPINB1] (26)	Multi-national	Periapical tissue biopsies	Case-control	141 (114 AP; 27 healthy)	Active/progressive vs. Stable/inactive apical periodontitis; Healthy periodontal ligament	2D-DIGE/MALDI-TOF MS (5800 spectrometer)	GPS Explorer v3.5 (MASCOT engine)	NR (≥95% CI)	NCBI non-redundant/ SwissProt	2D-DIGE fluorescence (Cy3/Cy5 labeling)
6	Wu et al. (2022) (27)	China	Periapical tissue	Comparative observational + *in vitro*	70 (23 AP; 47 PDL)	Chronic apical periodontitis vs. Healthy periodontal ligament; Rat experimental AP model	Label-free LC-MS/MS	NR	NR	NR	Label-free proteomics
7	Francisco et al. (2019) (28)	Brazil	Root canal contents	Cross-sectional (metaproteomics)	20 teeth	Post-treatment endodontic disease (persistent/secondary AP)	2D-LC-ESI-MS/MS (nano-flow)	Proteome Discoverer 1.3 (SEQUEST algorithm)	Decoy DB (reversed sequences)	Swiss-Prot + TrEMBL (Human + Microbial)	Spectral counting (frequency-based)
8	Nandakumar et al. (2009) (17)	USA	Root canal exudate (K-file + paper points)	Exploratory clinical (metaproteomics)	7	Endodontic infections (primary and persistent)	Shotgun LC-MS/MS (nano-flow reverse-phase)	SEQUEST (SORCERER IDA server)	NR (Xcorr >1.5/2.0/2.5; PeptideProphet >0.9)	NCBI bacterial DB (E. faecalis V583 + oral organisms)	Spectral counting (≥2 peptides/protein)
9	Provenzano et al. (2013) (16)	Brazil	Root canal/abscess fluid	Comparative observational	NR	Acute apical abscess vs. Asymptomatic apical periodontitis	Nanoflow LC-MS/MS	NR	NR	NR	Label-free/spectral counting
10	Jang et al. (2015) (29)	South Korea	Human dentin matrix	Cross-sectional (characterization)	3 (healthy 3rd molars)	Healthy dentin (baseline proteome characterization)	LC-MS/MS (LTQ Orbitrap)	SEQUEST (Sorcerer) + Scaffold	NR (≥95% peptide; ≥2 peptides/protein)	IPI Human protein database	Spectral counting (normalized)
11	Vidal et al. (2023) (30)	USA	Coronal dentin	Cross-sectional comparative	18 teeth (6 clusters)	Sound dentin vs. Distinct dentin caries (DDC) vs. Extensive dentin caries (EDC)	LC-MS/MS (label-free)	Proteome Discoverer (Mascot + Sequest HT)	FDR-adj. *p* < 0.05 (limma, Benjamini-Hochberg)	UniProt Homo sapiens	Label-free LFQ (Minora Feature Detection)

NR, not reported; FDR, false discovery rate; LC-MS/MS, liquid chromatography-tandem mass spectrometry. Search engine and FDR threshold data not consistently reported in primary studies; authors are requested to populate these columns from primary sources.

**Table 2:** Summary of included studies—proteomic findings.

#	Author (Year)	Total Proteins Identified (Human)	Key Biomarkers/DEPs	Enriched Pathways/Molecular Functions
1	Brizuela et al. (2025) (18)	577 proteins total; 139 common across groups; 82 unique to severe pulpitis	Hornerin↓ (severe vs. moderate); Cofilin-1↑; Peroxiredoxin-1↑; Haemoglobin *α*↑; FBPA↑; Calmodulin-like protein 5↓; Myosin light chain 6↓; S100-A8↑; S100-A9↑	Neutrophil degranulation; Antimicrobial defense; Energy metabolism; Structural integrity; Immune response escalation with severity
2	Silva et al. (2021) (21)	123 total: 66 healthy; 66 inflamed; 91 necrotic	Hb subunits↑ (×100 in inflamed); PRDX1/2↑ (×20); S100-A8/A9↑; Tubulin isoforms↓ (necrosis); Immunoglobulins↑; Serum albumin↑ (necrosis)	Oxidative stress response; Vascular response; Inflammation & immune activation; Cytoskeletal reorganization (necrosis); Multi-organism process
3	Silva et al. (2020) (25)	465 total (30 common to all groups)	Hb subunits↑ (inflamed); PRDX1/2↑; Tubulin↓ (17 isoforms, necrosis); Desmin↓; Alpha-internexin↓; Alpha-2-macroglobulin↑ (necrosis)	Oxidative stress; Cellular communication; Cytoskeletal destruction; Immune response (necrosis); Signal transduction
4	Loureiro et al. (2021) (22)	94 DEPs: 51↑ symptomatic; 43↓ symptomatic	MPO↑; S100-A8↑; Peroxiredoxin-1/2↑; Lactotransferrin↑; Alpha-1-antitrypsin↑; Defensins 1,3↓ (symptomatic); HSPs (HSPA1A/1B/6/8)↑ (asymptomatic exclusive); SOD↑ (symptomatic exclusive)	Neutrophil activation & NETs; ROS generation & antioxidant defense; Bone remodeling/osteoclast activity; Acute phase response
5	Cavalla et al. (2017) [HSP27/SERPINB1] (26)	48 DEPs: 30 in all lesions (19↓; 11↑)	HSP27↑ (3.79-fold, stable lesions); SERPINB1↑ (1.93-fold); MPO↑ (active lesions); MMP8↑ (active); Cathepsin G↑ (active); CXCR1↑ (active); HSP27 neg. corr. with MPO, CTSG, CXCR1	Apoptosis inhibition; Protease inhibition; Lesion stabilization vs. progression; Cytoskeletal remodeling; Neutrophil-mediated tissue damage
6	Wu et al. (2022) (27)	Not reported (targeted protein panel)	Caspase-1↑; Caspase-4↑; Caspase-5↑; GSDMD-N terminal domain↑; Mature IL-1β↑; CD68 + macrophages co-express caspase-5; Caspase-1/-11 dual role in bone loss	Canonical pyroptosis (Caspase-1/NLRP3); Non-canonical pyroptosis (Caspase-4/5); Inflammasome-mediated bone resorption; Double-edged sword: protective (small lesions), destructive (large lesions)
7	Francisco et al. (2019) (27)	1,153 human accessions; 720 microbial accessions; 11 human proteins in ≥35% cases	Hornerin↑; Mucin-5B↑; Mucin-16↑; Gremlin-1↑; TNF-R superfamily member 8↑; HLA-C↑; Microbial: TetM, VanSG2, Beta-lactamase, LPXTG-motif protein (Streptococcus anginosus)	Host-pathogen interaction; Antibiotic resistance mechanisms; Bone remodeling (Gremlin-1/BMP); Immune evasion; Biofilm maintenance
8	Nandakumar et al. (2009) (17)	Microbial-focused (bacterial proteome characterization)	E. faecalis: Gelatinase (GelE)↑; Ace↑; Cytolysin↑; Hemolysin A↑; VanSG2/VanE (vancomycin resistance); TetM (tetracycline resistance); Aggregation substance PrgB↑	Biofilm formation; ECM degradation; Antimicrobial resistance; Host cell invasion; Immune evasion; Bacterial adhesion & persistence
9	Provenzano et al. (2013) (16)	Microbial-focused (metaproteomics)	P. gingivalis gingipains↑; F. nucleatum adhesins↑; T. denticola virulence proteins↑; Polymicrobial virulence signature	Immune evasion; ECM degradation; Polymicrobial synergy; Host complement evasion; Differential expression: abscess vs. chronic AP
10	Jang et al. (2015) (29)	233 total (68 common to all 3 samples)	Collagens (I, III, V, VI, XI, XII); DSPP; DMP1; BSP2; Osteopontin; SPARC; Biglycan; Fibronectin; MMP20; TGF-β1; SOD3; PEDF; IGFBP-5	Dentin matrix organization; Dentinogenesis & mineralization; Odontoblast-related functions; Growth factor signaling; First comprehensive healthy dentin baseline
11	Vidal et al. (2023) (30)	320 total; 80 DEPs (EDC vs. Sound); 16 DEPs (EDC vs. DDC); 3 DEPs (DDC vs. Sound)	S100-A8↑; S100-A12↑; SERPINB1↑ (EDC); DSPP↓ (early caries); MMP20↑; DMP1↑; MPO↑; AZU1↑; LTF↑; PRTN3↑; TIMP-3↑; ENAM↑; CTHRC1↑; LOX↑	Neutrophil degranulation; NETs formation; Antimicrobial peptides; Dentin matrix degradation; Altered T & B cell signaling; Upstream regulators: CEBPA, IL1A, NFKBIZ

AP, apical periodontitis; DEPs, differentially expressed proteins; DMP1, dentin matrix protein 1; DSP, dentin sialoprotein; BSP, bone sialoprotein; ECM, extracellular matrix; GelE, gelatinase E; GSDMD, gasdermin D; Hb, hemoglobin; HSP27, heat shock protein 27; LC-MS/MS, liquid chromatography tandem mass spectrometry; MALDI-TOF MS, matrix-assisted laser desorption/ionization time-of-flight mass spectrometry; MPO, myeloperoxidase; NR, not reported; OPN, osteopontin; PRDX, peroxiredoxin; ROS, reactive oxygen species; SERPINB1, serpin family B member 1; SOD, superoxide dismutase; 2D-DIGE, two-dimensional difference gel electrophoresis.

**Table 3:** Risk of bias assessment of included studies using the modified QUADAS-2 tool.

Study	Patient Selection	Index Test	Reference Standard	Flow and Timing	Overall Risk of Bias
Loureiro et al., 2021 (22)	Low	Low	Low	Low	Low
Brizuela et al., 2025 (18)	Low	Low	Low	Low	Low
Francisco et al., 2019 (27)	Unclear	Low	Unclear	Unclear	Unclear
Silva et al., 2021 (21)	Low	Low	Low	Low	Low
Nandakumar et al., 2009 (17)	Unclear	Low	Unclear	Low	Unclear
Jang et al., 2015 (29)	Low	Low	Low	Low	Low
Silva et al., 2020 (25)	Unclear	Low	Unclear	Unclear	Unclear
Silva et al., 2021 (Clinical Stages) (21)	Low	Low	Low	Low	Low
Vidal et al., 2023 (30)	Low	Low	Low	Low	Low
Wu et al., 2022 (27)	Unclear	Low	Unclear	Low	Unclear
Cavalla et al., 2017 (26)	Low	Low	Low	Low	Low

Low risk (+); High risk (−); Unclear risk (?).

### 3.3 Proteomic profiling of pulpitis

Three studies specifically investigated proteomic changes across the spectrum of pulpitis from healthy pulp through reversible and irreversible pulpitis to pulp necrosis (18, 21, 25). Brizuela et al. (18) performed the most comprehensive analysis of dentinal fluid across pulpitis stages, identifying 577 proteins using state-of-the-art LC-MS/MS with label-free quantification on a timsTOF Pro 2 instrument. The study revealed progressive upregulation of inflammatory and stress response proteins with increasing disease severity. Hornerin (HRN), a keratin-associated protein not previously implicated in pulpal inflammation, emerged as a novel biomarker showing stage-dependent expression with highest levels in severe irreversible pulpitis. Other key differentially expressed proteins included calmodulin-like protein 5 (CALML5), cofilin-1 (CFL1), peroxiredoxin-1 (PRDX1), and fructose-bisphosphate aldolase A (ALDOA). Pathway enrichment analysis revealed significant activation of neutrophil degranulation, oxidative stress response, and glycolytic pathways in severe pulpitis, consistent with acute inflammatory processes and metabolic reprogramming (18).

Silva et al. (21) compared protein expression in healthy, inflamed, and necrotic dental pulp tissue from 36 patients, identifying 123 proteins distributed across 12 functional categories. The most striking finding was a 100-fold upregulation of hemoglobin subunits in inflamed pulp compared to healthy controls, indicating vascular disruption, hemorrhage, and red blood cell extravasation into the pulp chamber. Peroxiredoxins, key antioxidant enzymes that neutralize hydrogen peroxide and peroxynitrite, showed 20-fold upregulation in inflamed pulp, reflecting cellular responses to oxidative stress generated by inflammatory cells and bacterial products. S100-A8 and S100-A9 (calprotectin subunits) were markedly elevated in inflamed pulp, consistent with neutrophil infiltration and antimicrobial defense. Disease progression from inflammation to necrosis was characterized by a shift in proteomic profiles: inflamed pulp showed predominance of immune response and oxidative stress proteins, while necrotic pulp exhibited increased structural proteins (keratins), protein degradation products, and downregulation of metabolic enzymes, reflecting tissue breakdown and loss of cellular homeostasis (21).

A pilot study by Silva et al. (25) using nanoACQUITY UPLC-Xevo QTof MS identified 465 proteins in pulp tissue, confirming the hemoglobin and peroxiredoxin findings and additionally revealing downregulation of tubulin isoforms in necrotic pulp. Tubulins are essential cytoskeletal proteins required for cell division, intracellular transport, and maintenance of cell shape; their downregulation reflects cytoskeletal collapse and loss of cellular integrity during necrosis. Complement component C3 was upregulated in inflamed pulp, indicating activation of the complement cascade and opsonization of bacteria. These findings collectively demonstrate that pulpitis progression involves coordinated changes in vascular integrity, oxidative stress responses, immune activation, and ultimately cytoskeletal and metabolic collapse in necrotic tissue.

A cross-sectional study examining coronal dentin proteomics in sound teeth vs. teeth with dentin-dentin caries (DDC) and enamel-dentin caries (EDC) identified 320 proteins, with 80 differentially expressed in EDC compared to sound dentin. S100 family proteins (S100-A8, S100-A9, S100-A12) were markedly upregulated in carious dentin adjacent to inflamed pulp, suggesting that inflammatory mediators from the pulp diffuse through dentinal tubules and alter dentin matrix protein composition. SERPINB1, a serine protease inhibitor that protects tissues from neutrophil elastase and cathepsin G, was also upregulated, likely as a protective response to limit proteolytic damage. Dentin sialophosphoprotein (DSPP) and matrix metalloproteinase-20 (MMP20) showed altered expression, reflecting active matrix remodeling during caries progression. Pathway analysis revealed significant enrichment of the neutrophil degranulation pathway, indicating that innate immune responses extend from the inflamed pulp into the overlying dentin matrix.

### 3.4 Proteomic profiling of apical periodontitis

Four studies investigated proteomic signatures of apical periodontitis, comparing symptomatic vs. asymptomatic presentations, acute vs. chronic forms, and diseased vs. healthy periapical tissues (16, 22, 26, 27). Loureiro et al. (22) performed a landmark comparative proteomic analysis of root canal samples from patients with symptomatic vs. asymptomatic apical periodontitis using nLC-ESI-MS/MS. The study identified 51 up-regulated and 43 down-regulated proteins in symptomatic cases, revealing distinct molecular signatures associated with pain and acute inflammation. Alpha-1-antitrypsin, a serine protease inhibitor that regulates neutrophil elastase and protects tissues from proteolytic damage, was significantly upregulated in symptomatic apical periodontitis, suggesting an attempt to limit excessive tissue destruction. S100-A8 (calgranulin A), a calcium-binding protein released by neutrophils with antimicrobial and pro-inflammatory properties, was elevated in symptomatic cases, consistent with acute neutrophil infiltration (22).

Myeloperoxidase (MPO), a heme enzyme released by activated neutrophils that generates hypochlorous acid and other reactive oxygen species, was markedly upregulated in symptomatic apical periodontitis, indicating oxidative burst activity and potential for oxidative tissue damage. Peroxiredoxin-1 and peroxiredoxin-2, antioxidant enzymes that detoxify hydrogen peroxide and peroxynitrite, were also elevated, reflecting cellular attempts to counteract oxidative stress. Lactotransferrin (lactoferrin), an iron-binding glycoprotein with antimicrobial, anti-inflammatory, and immunomodulatory functions, was increased in symptomatic cases. Hemoglobin subunits (*β*, *ζ*, *α*, *δ*, *γ*, *ε*) were highly abundant in symptomatic apical periodontitis, indicating hemorrhage and red blood cell lysis in the periapical region. Interestingly, neutrophil defensin 1 and 3, antimicrobial peptides that kill bacteria through membrane disruption, were down-regulated in symptomatic cases, potentially reflecting defensin consumption during active infection or proteolytic degradation by bacterial proteases (22).

Proteins exclusive to symptomatic apical periodontitis included superoxide dismutase (SOD), an antioxidant enzyme that converts superoxide radicals to hydrogen peroxide, and several heat shock proteins (HSPA1A, HSPA1B, HSPA1L, HSPA2, HSPA4L, HSPA6, HSPA8). Heat shock proteins function as molecular chaperones that refold damaged proteins, prevent aggregation, and regulate apoptosis; their presence in symptomatic cases suggests cellular stress responses. Proteins exclusive to asymptomatic apical periodontitis included azurocidin (a neutrophil granule protein with antimicrobial and inflammatory properties), C-reactive protein (an acute phase reactant), collagen alpha chains (structural proteins), cathepsins (lysosomal proteases), and additional heat shock proteins. The presence of structural proteins and tissue repair markers in asymptomatic cases suggests a more chronic, stable lesion with ongoing remodeling rather than acute destruction. Components of neutrophil extracellular traps (NETs)—web-like structures composed of DNA, histones, and antimicrobial proteins released by neutrophils to trap and kill bacteria—were identified in both symptomatic and asymptomatic groups but with differential expression patterns, indicating that NET formation is a common feature of apical periodontitis but with quantitative differences related to disease activity (22).

Cavalla et al. (26) used 2D-DIGE coupled with MALDI-TOF MS/MS to identify 48 differentially expressed proteins in periapical tissue biopsies compared to healthy periodontal ligament. Two proteins emerged as particularly significant: heat shock protein 27 (HSP27, also known as HSPB1) was upregulated 3.79-fold, and serpin family B member 1 (SERPINB1, also known as MNEI or leukocyte elastase inhibitor) was upregulated 1.93-fold in apical periodontitis lesions. Immunofluorescence localization studies revealed that HSP27 was predominantly expressed in epithelial cells within periapical granulomas and cysts, while SERPINB1 was localized to both neutrophils and epithelial cells. Importantly, both HSP27 and SERPINB1 were significantly higher in stable or inactive lesions compared to active, progressing lesions (*p* < 0.05), suggesting protective roles in limiting tissue damage and promoting lesion stabilization (26).

HSP27 functions as a molecular chaperone that stabilizes partially denatured proteins, inhibits apoptosis by interfering with cytochrome c release and caspase activation, and regulates actin cytoskeleton dynamics. Its upregulation in stable lesions suggests a tissue-protective role during chronic inflammation, preventing excessive cell death and maintaining tissue architecture. SERPINB1 protects tissues against neutrophil-derived serine proteases including elastase, cathepsin G, and proteinase-3, which can cause collateral tissue damage during inflammation. By inhibiting these proteases, SERPINB1 limits proteolytic destruction of extracellular matrix and prevents excessive tissue breakdown. Correlation analysis revealed significant negative correlations between HSP27 and inflammatory markers including CXCR1 (IL-8 receptor, r^2^ = 0.12, *p* < 0.001), myeloperoxidase (MPO, r^2^ = 0.06, *p* = 0.007), and cathepsin G (CTSG, r^2^ = 0.08, *p* = 0.002), supporting an anti-inflammatory function. Similarly, SERPINB1 showed negative correlations with CXCR1 (r^2^ = 0.09, *p* = 0.001) and MMP8, further supporting its protective role (26).

A recent study (27) employed label-free proteomics to investigate pyroptosis pathway activation in apical periodontitis tissues. Pyroptosis is a form of inflammatory programmed cell death mediated by caspases-1, -4, -5, and -11 (in mice) that cleave gasdermin D (GSDMD) to generate an N-terminal fragment (GSDMD-N) that oligomerizes and forms pores in the plasma membrane, causing cell lysis and release of inflammatory cytokines. The study identified significant upregulation of caspase-1, caspase-4, and caspase-5 in periapical lesions, along with GSDMD and mature interleukin-1β (IL-1β). Pyroptosis serves a “double-edged sword” function: it eliminates infected cells and releases alarmins that activate immune responses, but excessive pyroptosis causes collateral tissue damage, amplifies inflammation, and promotes bone resorption through IL-1β-mediated osteoclast activation. The findings suggest that pyroptosis inhibitors might represent novel therapeutic targets for limiting periapical bone destruction while preserving antimicrobial defenses.

### 3.5 Proteomic profiling of post-treatment endodontic disease

Francisco et al. (27) conducted the first comprehensive metaproteomic analysis of root canal contents in teeth with post-treatment endodontic disease (persistent or secondary apical periodontitis following root canal treatment). Using two-dimensional liquid chromatography coupled to electrospray ionization tandem mass spectrometry (2D-LC-ESI-MS/MS), the study identified an unprecedented 1,153 human proteins and 720 microbial proteins, providing the most comprehensive molecular characterization of treatment-resistant endodontic infections to date. Among human proteins, hornerin was highly abundant, consistent with findings in primary infections and suggesting a conserved host response. Mucin-5B, a large gel-forming mucin that contributes to mucosal barrier function and innate immunity, was also prominently detected, potentially reflecting epithelial responses in the root canal system or contamination from oral fluids (3).

Gremlin-1, a bone morphogenetic protein (BMP) antagonist that inhibits bone formation and promotes osteoclast differentiation, was identified in post-treatment disease, suggesting a molecular mechanism for persistent periapical bone resorption despite treatment. TNF receptor superfamily member 8 (TNFRSF8, also known as CD30), a receptor involved in immune regulation and cell survival, was detected, indicating ongoing immune activation. Immunoglobulins and keratins were abundant, consistent with chronic inflammation and epithelial involvement. S100 proteins were again prominent, reinforcing their role as core biomarkers across the spectrum of endodontic diseases (3).

The microbial metaproteome revealed a complex polymicrobial community dominated by *E. faecalis*, *P. gingivalis*, *F. nucleatum*, *T. forsythia*, and *P. endodontalis*. Virulence factors identified included adhesins that mediate bacterial attachment to dentin and biofilm formation, proteases (gingipains from *P. gingivalis*, gelatinase from *E. faecalis*) that degrade host proteins and extracellular matrix, toxins (cytolysin from *E. faecalis*) that lyse host cells, and antibiotic resistance proteins including beta-lactamases and efflux pumps. The presence of antibiotic resistance determinants provides molecular evidence for why systemic antibiotics often fail to resolve post-treatment endodontic disease. The study demonstrated that post-treatment infections involve complex host-pathogen interaction networks with synergistic virulence mechanisms, where multiple species cooperate to resist host defenses and endodontic treatments (27).

### 3.6 Microbial proteomics in endodontic infections

Nandakumar et al. (17) pioneered the application of shotgun proteomics to identify microbial virulence factors in endodontic infections, focusing particularly on *E.faecalis*, a Gram-positive bacterium frequently isolated from treatment-resistant root canal infections. Using LC-MS/MS analysis of root canal specimens from 15 patients, the study identified key enterococcal virulence proteins including adhesins (Ace, EfaA), autolysins (AtlA), gelatinase (GelE), and cytolysin (CylA). Ace (adhesin to collagen of *E. faecalis*) and EfaA (endocarditis and biofilm-associated pilus protein A) mediate bacterial attachment to dentin collagen and facilitate biofilm formation, enabling persistent colonization of the root canal system. AtlA (autolysin A) is involved in cell wall turnover, biofilm formation, and release of extracellular DNA that stabilizes biofilms (17).

Gelatinase (GelE) is a zinc metalloprotease that degrades gelatin, collagen, fibrin, and other extracellular matrix proteins, contributing to tissue invasion and immune evasion by cleaving complement components and antimicrobial peptides. Cytolysin (CylA) is a pore-forming toxin that lyses host cells including neutrophils, macrophages, and epithelial cells, causing tissue damage and releasing nutrients for bacterial growth. The study also identified antibiotic resistance proteins including TetM (conferring tetracycline resistance through ribosomal protection) and VanSG2 (component of vancomycin resistance systems), providing molecular explanations for treatment failures with these antibiotics. In addition to *E. faecalis*, the study detected proteins from *P. gingivalis*, *F. nucleatum*, and *T. forsythia*, demonstrating polymicrobial communities in endodontic infections. *F. nucleatum* serves as a bridging organism that co-aggregates with multiple species and enhances biofilm formation, while *P. gingivalis* produces potent proteases (gingipains) that degrade host proteins and dysregulate immune responses (17).

Provenzano et al. (16) extended microbial proteomic analysis to compare acute apical abscesses vs. asymptomatic apical periodontitis, using complementary mass spectrometry platforms (nanoflow LC-Orbitrap Velos and LC-QTOF). The study identified species-specific virulence factors and revealed that acute abscesses harbor more diverse and virulent microbial communities compared to asymptomatic lesions. Glycosyltransferases involved in biofilm matrix synthesis were abundant, indicating that biofilm formation is a key survival strategy in both acute and chronic infections. Proteases, including gingipains, were more abundant in acute abscesses, consistent with active tissue destruction. The study demonstrated that metaproteomics can distinguish microbial community structures and virulence profiles associated with different clinical presentations, potentially guiding targeted antimicrobial therapies (16).

### 3.7 Dentin matrix proteomics

Jang et al. (29) performed comprehensive proteomic characterization of healthy human dentin matrix using LC-MS/MS, identifying 233 proteins that constitute the organic component of dentin. Collagens, particularly type I collagen (COL1A1, COL1A2), comprised the majority of the dentin matrix, providing structural scaffolding and tensile strength. Additional collagen types identified included types III, V, VI, XI, and XII, which regulate fibril assembly, interact with other matrix components, and modulate tissue mechanical properties. Non-collagenous proteins (NCPs) identified included dentin sialophosphoprotein (DSPP), which is cleaved into dentin sialoprotein (DSP) and dentin phosphoprotein (DPP) that regulate hydroxyapatite crystal nucleation and growth during mineralization (29).

Dentin matrix protein 1 (DMP1), a member of the SIBLING (Small Integrin-Binding Ligand, N-linked Glycoprotein) family, was detected and functions in mineralization regulation and osteoblast/odontoblast differentiation. Osteopontin (SPP1), another SIBLING family member, regulates cell adhesion, migration, and mineralization. SPARC (secreted protein acidic and rich in cysteine, also known as osteonectin) modulates collagen fibrillogenesis and cell-matrix interactions. Biglycan (BGN), a small leucine-rich proteoglycan, regulates collagen fibril assembly and growth factor signaling. Matrix metalloproteinase-20 (MMP20, enamelysin) was identified, suggesting roles in matrix remodeling during dentinogenesis. Transforming growth factor-*β*1 (TGF*β*1), a pleiotropic cytokine that regulates cell proliferation, differentiation, and extracellular matrix production, was embedded in the dentin matrix, potentially serving as a reservoir released during caries or trauma. Superoxide dismutase 3 (SOD3), an extracellular antioxidant enzyme, was also detected, suggesting that dentin matrix contains protective factors against oxidative stress (29).

This comprehensive dentin proteome provides a baseline for understanding how protein composition changes during caries, pulpal inflammation, and reparative dentinogenesis. The presence of growth factors and bioactive molecules embedded in the dentin matrix suggests that dentin is not merely a structural tissue but an active reservoir of signaling molecules that can be released during disease or treatment (e.g., during cavity preparation or acid etching) to influence pulpal responses. Understanding the dentin proteome is essential for interpreting proteomic changes in dentinal fluid and pulp tissue during disease progression (29).

## 4 Discussion

### 4.1 Proteomic signatures across pulpitis stages

The proteomic studies reviewed herein reveal distinct molecular signatures that characterize the progression from healthy dental pulp through reversible pulpitis, irreversible pulpitis, and ultimately pulp necrosis. These stage-specific proteomic profiles reflect the underlying pathophysiological transitions from initial inflammatory responses to overwhelming tissue destruction and loss of cellular homeostasis. The identification of hornerin as a novel biomarker in dentinal fluid represents a significant advance, as this keratin-associated protein has not been previously implicated in pulpal pathology (18). Hornerin is normally expressed in epithelial tissues undergoing terminal differentiation and cornification, and its presence in dentinal fluid during pulpitis may reflect epithelial metaplasia, odontoblast stress responses, or infiltration of oral epithelial cells through carious lesions. The progressive upregulation of hornerin from mild to severe pulpitis suggests potential utility as a quantitative biomarker for disease severity, warranting validation in larger cohorts and investigation of its functional role in pulpal inflammation.

The 100-fold upregulation of hemoglobin subunits in inflamed pulp tissue reflects vascular disruption, increased permeability, and red blood cell extravasation—hallmarks of acute inflammation (21, 25). Extracellular hemoglobin generates reactive oxygen species, amplifies oxidative tissue damage, and provides an iron source for bacterial growth, while concurrent upregulation of scavenger proteins (haptoglobin, hemopexin) reflects partial protective compensation (30, 31). Clinically, hemoglobin levels in dentinal fluid or pulpal blood sampled from cavity preparations may serve as accessible chairside indicators of pulpal inflammation severity.

Peroxiredoxins (PRDX1, PRDX2) were consistently upregulated across multiple pulpitis studies, showing up to 20-fold increases in inflamed pulp (18, 21, 25). Their elevation reflects cellular antioxidant responses to oxidative stress generated by neutrophil respiratory burst, bacterial metabolic products, and mitochondrial dysfunction in stressed pulp cells. Consistent detection of peroxiredoxins across different specimen types (pulp tissue, dentinal fluid) and analytical platforms strengthens their candidacy as core biomarkers of pulpal oxidative stress.

S100 family proteins, particularly S100-A8 and S100-A9 (calprotectin heterodimer), were prominently upregulated in inflamed pulp and dentinal fluid across multiple studies (18, 21). Released by activated neutrophils and monocytes, these DAMPs amplify inflammation and sequester metal ions (zinc, manganese) required by bacteria, linking host inflammatory signaling to direct antimicrobial activity (33, 34). Their consistent identification across diverse specimen types—pulp tissue, dentinal fluid, and root canal exudates—establishes them as robust and reproducible candidate biomarkers of pulpal and periapical inflammation.

The shift in proteomic profiles from inflamed to necrotic pulp reveals fundamental changes in tissue biology. Inflamed pulp is characterized by active immune responses (immunoglobulins, complement components), oxidative stress responses (peroxiredoxins, superoxide dismutase), and vascular changes (hemoglobin), reflecting viable cells mounting defensive responses (35). In contrast, necrotic pulp shows downregulation of metabolic enzymes, cytoskeletal proteins (tubulins, actins), and stress response proteins, with increased abundance of structural proteins (keratins) and protein degradation products, reflecting loss of cellular integrity and tissue breakdown (25). This transition from active inflammation to necrosis has important clinical implications: inflamed but viable pulp may be amenable to vital pulp therapy and regenerative approaches, whereas necrotic pulp requires complete debridement and root canal treatment. Proteomic biomarkers that accurately distinguish these states could guide treatment decisions and improve outcomes.

### 4.2 Biomarkers of apical periodontitis

Proteomic profiling of apical periodontitis has revealed distinct molecular signatures distinguishing symptomatic from asymptomatic presentations, acute from chronic forms, and active from stable lesions, providing molecular explanations for clinical heterogeneity. The identification of 51 upregulated and 43 downregulated proteins in symptomatic vs. asymptomatic apical periodontitis represents the most comprehensive comparative analysis to date (22). Myeloperoxidase (MPO), prominently upregulated in symptomatic cases, generates hypochlorous acid that kills bacteria but also oxidatively activates nociceptors, providing a direct molecular basis for pain in acute presentations (36). MPO-derived oxidants further modify host proteins, generating neo-antigens that perpetuate local inflammation.

Alpha-1-antitrypsin, upregulated in symptomatic apical periodontitis, reflects a host attempt to limit neutrophil-mediated proteolytic destruction (22). The balance between neutrophil elastase and its inhibitors (alpha-1-antitrypsin, SERPINB1) critically determines the extent of periapical tissue and bone destruction; an insufficient antiprotease response may drive aggressive bone loss, while effective inhibition supports lesion containment. This protease-antiprotease axis is a compelling therapeutic target in symptomatic acute presentations.

The downregulation of neutrophil defensins 1 and 3 in symptomatic apical periodontitis—despite active neutrophil infiltration—is counterintuitive and potentially significant (22). Possible explanations include consumption during intense antimicrobial activity, proteolytic degradation by bacterial enzymes (gingipains, gelatinase), or impaired degranulation under conditions of overwhelming bacterial challenge. Whether defensin deficiency reflects or actively contributes to symptom severity warrants longitudinal investigation.

HSP27 and SERPINB1 upregulation in stable vs. active periapical lesions provides important mechanistic insight into lesion stabilization (26). HSP27 functions as a molecular chaperone and anti-apoptotic regulator that maintains cellular homeostasis; its elevation in stable lesions and negative correlation with inflammatory markers (CXCR1, MPO, cathepsin G) supports an active tissue-protective role. These findings identify HSP27 as both a prognostic biomarker of lesion stability and a potential therapeutic target.

SERPINB1, an intracellular inhibitor of neutrophil serine proteases (elastase, cathepsin G, proteinase-3), complements HSP27 in limiting neutrophil-mediated tissue destruction in stable lesions (37, 38). Together, elevated HSP27 and SERPINB1 represent a molecular signature of lesion equilibrium, suggesting that strategies to pharmacologically enhance their expression might promote stabilization and healing of active periapical lesions.

The identification of pyroptosis pathway components (caspases-1/-4/-5, GSDMD, IL-1β) in apical periodontitis specimens represents a paradigm shift in understanding periapical bone destruction mechanisms (27). Pyroptosis—inflammatory programmed cell death triggered by bacterial PAMPs—eliminates infected cells while releasing pro-inflammatory mediators that amplify bone resorption, directly linking microbial infection to osteolysis (39).

The “double-edged sword” nature of pyroptosis captures this dual role: beneficial bacterial clearance vs. collateral amplification of IL-1β-driven RANKL expression and osteoclastogenesis (27, 40). This axis suggests pyroptosis inhibitors or GSDMD-targeted agents as novel adjunctive strategies to limit bone destruction, though careful titration is needed to preserve antimicrobial defenses.

### 4.3 Post-Treatment endodontic disease proteins

The comprehensive metaproteomic analysis of post-treatment endodontic disease by Francisco et al. (27) provides unprecedented insights into the molecular mechanisms underlying treatment failure and persistent periapical pathology. The identification of 1,153 human proteins and 720 microbial proteins represent the most extensive molecular characterization of treatment-resistant infections to date, revealing complex host-pathogen interaction networks that explain why some cases fail to heal despite technically adequate root canal treatment. The prominence of hornerin in post-treatment disease, consistent with its identification in primary infections, suggests that this protein represents a conserved host response to endodontic infections regardless of treatment history (18, 27). Further research is needed to determine whether hornerin levels correlate with treatment outcomes and whether it serves protective or pathological functions.

Mucin-5B abundance in post-treatment disease likely reflects either contamination through inadequate coronal seal or production by epithelial cells in communicating periapical cysts (27). Its potential role as a bacterial substrate sustaining persistent microorganisms adds clinical significance, warranting targeted study of its contribution to treatment failure.

Gremlin-1—a BMP antagonist that inhibits osteoblast differentiation and independently promotes osteoclastogenesis—was identified in post-treatment disease, offering a molecular explanation for persistent bone resorption despite reduced bacterial load (27, 41). Therapeutic neutralization of Gremlin-1 or augmentation of BMP signaling may promote periapical bone regeneration in refractory cases, representing a novel non-antimicrobial treatment strategy.

The post-treatment microbial metaproteome was dominated by *E. faecalis, P. gingivalis*, *F. nucleatum*, *T. forsythia*, and *P. endodontalis*, with extensive virulence factor expression (17, 27). Proteomic identification of *E. faecalis* adhesins (Ace, EfaA), gelatinase (GelE), and cytolysin (CylA) provides molecular confirmation of the multi-factorial mechanisms underlying its persistence in the root canal—biofilm formation, tissue invasion, and direct cytotoxicity.

Detection of antibiotic resistance determinants—TetM (tetracycline), VanSG2 (vancomycin), and beta-lactamases—in post-treatment infections provides molecular evidence for the inadequacy of systemic antibiotic monotherapy in most endodontic cases (17, 27). These findings reinforce mechanical debridement and antiseptic irrigation as the primary treatment modality and underscore the need for novel strategies targeting resistant biofilms.

### 4.4 Host-Pathogen interactions

The proteomic studies reviewed herein provide molecular evidence for complex host-pathogen interactions that determine the outcome of endodontic infections. The simultaneous identification of host and microbial proteins in the same specimens enables reconstruction of interaction networks and identification of key molecular interfaces. *F. nucleatum* emerged as a keystone pathogen in multiple studies, serving as a bridging organism that co-aggregates with diverse bacterial species and enhances biofilm formation (16, 17). *F. nucleatum* expresses numerous adhesins including FadA (Fusobacterium adhesin A) that bind to host cells and other bacteria, facilitating polymicrobial community assembly. It also produces butyrate, a short-chain fatty acid that inhibits histone deacetylases and modulates host gene expression, potentially suppressing immune responses and promoting bacterial survival (42).

*P. gingivalis*—identified in both primary and post-treatment endodontic infections—employs cysteine proteases (gingipains: Rgp, Kgp) to degrade immunoglobulins, complement, and antimicrobial peptides, facilitating immune evasion (43). Its intracellular invasion of epithelial cells and TLR4 antagonism further explain its persistence in the root canal system, contributing to cases of refractory apical periodontitis that remain challenging to manage clinically.

NET components—myeloperoxidase, neutrophil elastase, cathepsin G, and defensins—identified in apical periodontitis specimens provide direct evidence for active NETosis in endodontic infections (22). While NETs are critical for trapping and killing bacteria, uncontrolled NET formation also mediates collateral tissue damage through histonecytotoxicity and matrix-degrading proteases, potentially amplifying periapical bone loss in severe or chronic cases.

Some bacteria have evolved mechanisms to evade or degrade NETs, contributing to persistent infection. *P. gingivalis* gingipains degrade NET components, enabling bacterial escape (44). *E. faecalis* produces nucleases that degrade NET DNA, disrupting the web structure (45). The balance between NET formation and bacterial NET evasion likely determines infection outcomes. Therapeutic strategies to enhance NET formation or prevent NET degradation might improve bacterial clearance, while strategies to limit excessive NET formation might reduce tissue damage. The differential expression of NET components between symptomatic and asymptomatic apical periodontitis suggests that NET biology influences clinical presentation (22).

### 4.5 Molecular pathways in endodontic diseases

Neutrophil degranulation was the most consistently enriched pathway across studies (18, 30). Proteomic identification of granule marker proteins—myeloperoxidase, lactoferrin, MMP-9, and alkaline phosphatase—documents complete sequential degranulation and confirms that the full antimicrobial neutrophil program is activated in endodontic infections (46). Beyond neutrophil pathways, oxidative stress response, inflammatory cytokine signaling, pyroptosis, bone remodeling, and extracellular matrix degradation were consistently activated across studies, forming an integrated molecular network of endodontic pathology.

Nrf2-mediated oxidative stress response pathways were activated across pulpitis stages and apical periodontitis, evidenced by consistent upregulation of Nrf2 target genes—peroxiredoxins, superoxide dismutases, catalase (18, 21). However, the simultaneous detection of oxidative damage markers indicates that antioxidant defenses are ultimately overwhelmed in progressive disease, contributing to tissue destruction and underscoring oxidative stress as a therapeutic target (46).

Inflammatory signaling pathways—NF-*κ*B, MAPK, and JAK-STAT—were activated across studies, driving pro-inflammatory cytokine production, immune cell recruitment, and perpetuation of chronic periapical pathology (16, 27). Their consistent proteomic signature confirms that canonical innate immune cascades are fully engaged throughout the disease spectrum (22).

The RANKL-RANK-OPG bone remodeling axis was activated in periapical disease, with inflammatory cytokines shifting the RANKL/OPG balance toward osteoclastogenesis and bone resorption (26, 27, 39). Proteomic identification of RANKL, OPG, and related mediators in periapical tissue provides direct molecular evidence for this mechanism and identifies the RANKL/OPG ratio as a potential prognostic biomarker for bone loss severity.

MMPs and TIMPs regulate extracellular matrix degradation in endodontic diseases; neutrophil-derived MMP-8 and MMP-9 mediate periapical tissue and bone destruction, while MMP-20 participates in dentin matrix remodeling (29, 30, 48) (Stage-Specific Study, 2023). An imbalanced MMP/TIMP ratio, favoring excessive proteolysis, represents a mechanistic driver of pathological tissue loss and a potential therapeutic target for limiting periapical bone destruction.

### 4.6 Clinical translation and biomarker applications

The proteomic biomarkers identified in this systematic review hold significant promise for development of diagnostic and prognostic tools that could transform endodontic practice. Current diagnostic methods rely on subjective symptom assessment, clinical tests of limited sensitivity and specificity (percussion, palpation, thermal testing), and radiographic evaluation that detects only advanced bone loss (4). Molecular biomarkers could provide objective, quantitative measures of disease presence, severity, and activity, enabling earlier diagnosis, more accurate treatment planning, and prediction of treatment outcomes. Several biomarkers identified in this review are particularly promising for clinical translation.

Hornerin in dentinal fluid represents an attractive diagnostic biomarker due to its progressive upregulation across pulpitis stages, accessibility of dentinal fluid sampling, and potential for immunoassay-based detection (18). Dentinal fluid can be collected non-invasively from cavity preparations or through minimally invasive dentin sampling, making it suitable for chairside testing. An immunoassay (ELISA or lateral flow device) targeting hornerin could provide rapid, quantitative assessment of pulpal inflammation severity, guiding decisions between vital pulp therapy and pulpectomy. Validation studies are needed to establish hornerin cutoff values that distinguish reversible from irreversible pulpitis with high sensitivity and specificity, and to determine whether hornerin levels predict treatment outcomes.

S100 proteins (S100-A8, S100-A9, S100-A12) are strong biomarker candidates due to their consistent upregulation across multiple studies, well-established roles in inflammation, and availability of validated immunoassays (18, 21, 30). Calprotectin (S100-A8/A9 heterodimer) is already used clinically as a fecal biomarker for inflammatory bowel disease, demonstrating feasibility of S100-based diagnostics (49). In endodontics, S100 proteins could be measured in dentinal fluid, root canal exudates, or periradicular tissue fluid to assess inflammatory activity. Elevated S100 levels might predict symptomatic presentations, guide antibiotic use in acute infections, or indicate need for retreatment in persistent apical periodontitis. Longitudinal studies measuring S100 levels before and after treatment could determine whether declining levels correlate with healing.

HSP27 and SERPINB1 in periapical tissues show promise as prognostic biomarkers for predicting lesion behavior and treatment outcomes (26). The finding that these proteins are elevated in stable vs. active lesions suggests they could identify cases likely to heal vs. those requiring surgical intervention. Periradicular tissue fluid sampling through the root canal or via aspiration could enable minimally invasive measurement of HSP27 and SERPINB1 levels. High levels might predict favorable healing, while low levels might indicate need for more aggressive treatment or adjunctive therapies. Alternatively, these proteins could serve as therapeutic targets: strategies to enhance HSP27 or SERPINB1 expression (e.g., through pharmacological chaperone inducers or gene therapy) might promote lesion stabilization and healing.

MPO in root canal exudates or periradicular tissue fluid could reliably predict symptomatic presentations, guide pain management decisions, and identify cases requiring emergency treatment (22, 26). Given existing validated MPO immunoassays already used in clinical diagnostics, translation to chairside endodontic testing is technically straightforward and warrants prospective validation against clinical outcomes.

Pyroptosis pathway components (caspases-1/-4/-5, GSDMD, IL-1β) represent potential prognostic biomarkers for periapical bone destruction and treatment outcomes (27). High levels of pyroptosis markers might predict aggressive bone resorption and poor healing, identifying cases that would benefit from adjunctive anti-inflammatory therapies or caspase inhibitors. Conversely, low pyroptosis activity might indicate chronic, stable lesions with better prognosis. IL-1β is measurable in various biological fluids using ELISA, and caspase activity can be assessed using fluorogenic substrates. Longitudinal studies correlating pyroptosis biomarker levels with radiographic bone healing outcomes are needed to validate their prognostic utility.

Microbial virulence factors identified through metaproteomics could guide targeted antimicrobial therapies (17, 27). Detection of specific virulence proteins (e.g., enterococcal gelatinase, cytolysin; P. gingivalis gingipains) could indicate presence of particularly virulent strains requiring aggressive treatment. Identification of antibiotic resistance proteins (TetM, VanSG2, beta-lactamases) could guide antibiotic selection in cases requiring systemic antimicrobial therapy, avoiding ineffective agents. Metaproteomic profiling could also identify polymicrobial communities with synergistic virulence, indicating need for combination antimicrobial approaches. However, the complexity and cost of mass spectrometry-based metaproteomics currently limit clinical application; development of targeted assays for key virulence factors would facilitate translation.

### 4.7 Current Challenges and Limitations of Proteomic and Metaproteomic Research

Despite significant advances, proteomic and metaproteomic studies in endodontics continue to face several methodological and technical challenges that limit their clinical translation. Most included studies involved relatively small sample sizes, reducing statistical power and limiting the generalizability of identified biomarkers. Variability in sample collection, storage, protein extraction, and analytical workflows further complicates comparison across studies and highlights the need for standardized operating protocols.

Another major limitation is the wide dynamic range of protein concentrations in biological samples. Highly abundant proteins, including albumin and immunoglobulins, frequently mask low-abundance regulatory proteins and inflammatory mediators that may serve as clinically relevant biomarkers. Notably, none of the included studies employed high-abundance protein depletion strategies before LC-MS/MS analysis, potentially limiting proteome depth and reducing the detection of disease-specific proteins.

Microbial metaproteomics presents an additional bioinformatics challenge. Protein identification currently relies heavily on homology-based searches against incomplete and poorly annotated oral bacterial genome databases. Because many periodontal and endodontic microorganisms within complex polymicrobial biofilms remain only partially sequenced, species-specific virulence factors are frequently under-identified or incorrectly assigned through cross-species homology. This bioinformatics bottleneck limits accurate attribution of pathogenic protein expression to individual bacterial species and reduces the biological interpretation of microbial proteomic datasets. Expansion of comprehensive oral microbial genome databases, improved functional protein annotation, and development of advanced bioinformatic pipelines are therefore essential for improving the accuracy and clinical utility of microbial metaproteomic analyses.

Most studies employed discovery-phase proteomics without independent validation in separate cohorts. Rigorous biomarker validation using orthogonal methods such as immunoassays or targeted mass spectrometry is essential to confirm differential protein expression and establish diagnostic and prognostic performance before clinical implementation. Longitudinal studies correlating baseline proteomic profiles with treatment outcomes are also lacking. Prospective investigations are required to determine whether identified biomarkers can predict disease progression, healing, recurrence, and long-term treatment success. Finally, functional validation of candidate proteins remains limited. Although proteomic analyses identify differentially expressed proteins, they do not establish causal roles in disease pathogenesis. Future *in vitro*, *ex vivo*, and *in vivo* studies are necessary to determine the biological functions of these proteins and evaluate their potential as therapeutic targets.

### 4.8 Future directions

Future research should address the limitations identified above and advance proteomic profiling of endodontic diseases toward clinical translation. Large-scale, multi-center studies with standardized protocols are needed to validate candidate biomarkers in diverse patient populations. Such studies should employ rigorous study designs with adequate sample sizes, independent discovery and validation cohorts, and blinded outcome assessment. Standardization of pre-analytical protocols including sample collection, processing, storage, and protein extraction is essential for generating reproducible data. Development of consensus SOPs through professional organizations (American Association of Endodontists, European Society of Endodontology) would facilitate harmonization across studies.

Integration of proteomics with other omics technologies (genomics, transcriptomics, metabolomics, microbiomics) would provide systems-level understanding of endodontic disease mechanisms. Multi-omics approaches can identify regulatory networks, causal relationships, and therapeutic targets that are not apparent from single-platform studies. For example, integrating proteomic data with microbiome sequencing could correlate specific bacterial species or community structures with host proteomic responses, identifying key host-pathogen interactions. Integrating proteomics with metabolomics could reveal metabolic pathways altered during disease and identify metabolite biomarkers that complement protein biomarkers. Realising the full translational potential of such integrative analyses will additionally require advanced computational frameworks; in particular, multi-block partial least squares (MB-PLS) regression and related latent variable methods provide a statistically principled means of simultaneously decomposing shared variance across large-scale LC-MS/MS proteomics, metabolomics, and transcriptomics datasets, thereby enabling the identification of coherent cross-omic biomarker signatures for pulpal and periapical disease diagnosis and prognosis.

Development of targeted proteomic assays for validated biomarkers would facilitate clinical translation. While discovery proteomics using LC-MS/MS is powerful for unbiased protein identification, it is complex, expensive, and time-consuming, limiting clinical applicability. Targeted approaches including selected reaction monitoring (SRM) and parallel reaction monitoring (PRM) offer high sensitivity, specificity, and reproducibility for measuring pre-defined protein panels (14). Development of SRM/PRM assays for key biomarkers (hornerin, S100 proteins, HSP27, SERPINB1, MPO) would enable rapid, quantitative measurement in clinical specimens. Alternatively, immunoassays (ELISA, multiplex bead arrays, lateral flow devices) could provide point-of-care testing capabilities for chairside diagnostics.

Longitudinal studies correlating proteomic biomarkers with treatment outcomes are essential for identifying prognostic markers. Such studies should measure biomarkers at baseline (pre-treatment), during treatment (e.g., after instrumentation, after medication), and at follow-up intervals (3, 6, 12, 24 months), correlating levels with clinical and radiographic healing outcomes. Machine learning approaches could integrate multiple biomarkers with clinical variables (tooth type, lesion size, treatment complexity) to develop predictive models for treatment success. Identification of biomarkers that predict healing would enable personalized treatment planning, guiding decisions about treatment aggressiveness, adjunctive therapies, and follow-up intervals.

Investigation of post-translational modifications (PTMs) would provide deeper insights into protein function and regulation. PTMs including phosphorylation, acetylation, ubiquitination, glycosylation, and oxidative modifications regulate protein activity, localization, and interactions. Inflammatory signaling pathways are extensively regulated by phosphorylation, and oxidative stress causes protein carbonylation, nitration, and disulfide bond formation. Proteomic approaches that enrich and identify PTMs (phosphoproteomics, glycoproteomics, redox proteomics) could reveal regulatory mechanisms and identify modified proteins as biomarkers. For example, oxidatively modified proteins might serve as biomarkers of oxidative stress severity in pulpitis.

Development of proteomic-guided therapeutic strategies represents an exciting frontier. Identification of druggable targets through proteomics could enable development of targeted therapies for endodontic diseases. For example, pyroptosis inhibitors targeting caspases or GSDMD might limit periapical bone destruction (27). HSP27 inducers or SERPINB1 mimetics might promote lesion stabilization (16). Protease inhibitors targeting bacterial virulence factors (gingipains, gelatinase) might enhance treatment outcomes. Proteomic profiling could also guide patient stratification for clinical trials, identifying subgroups likely to respond to specific interventions based on their molecular profiles.

Spatial proteomics approaches including imaging mass spectrometry (MALDI imaging, DESI imaging) could map protein distributions within tissues, revealing spatial organization of inflammatory processes, bacterial colonization patterns, and tissue remodeling zones. Such approaches could distinguish protein expression in different tissue compartments (pulp chamber vs. root canal, epithelium vs. connective tissue in periapical lesions, infected vs. uninfected dentin) and identify spatial biomarkers. Single-cell proteomics, an emerging technology, could resolve cellular heterogeneity and identify cell type-specific responses to infection and inflammation.

Finally, translation of proteomic discoveries into clinical practice requires demonstration of clinical utility—that biomarker-guided decisions improve patient outcomes compared to standard care. This requires prospective randomized controlled trials comparing biomarker-guided treatment strategies vs. conventional approaches, with outcomes including healing rates, symptom resolution, quality of life, and cost-effectiveness. Such trials are resource-intensive but essential for establishing clinical value and achieving adoption by practitioners and reimbursement by payers. Collaboration between researchers, clinicians, industry partners, and regulatory agencies will be necessary to navigate the pathway from biomarker discovery to clinical implementation.

**Figure 2:**
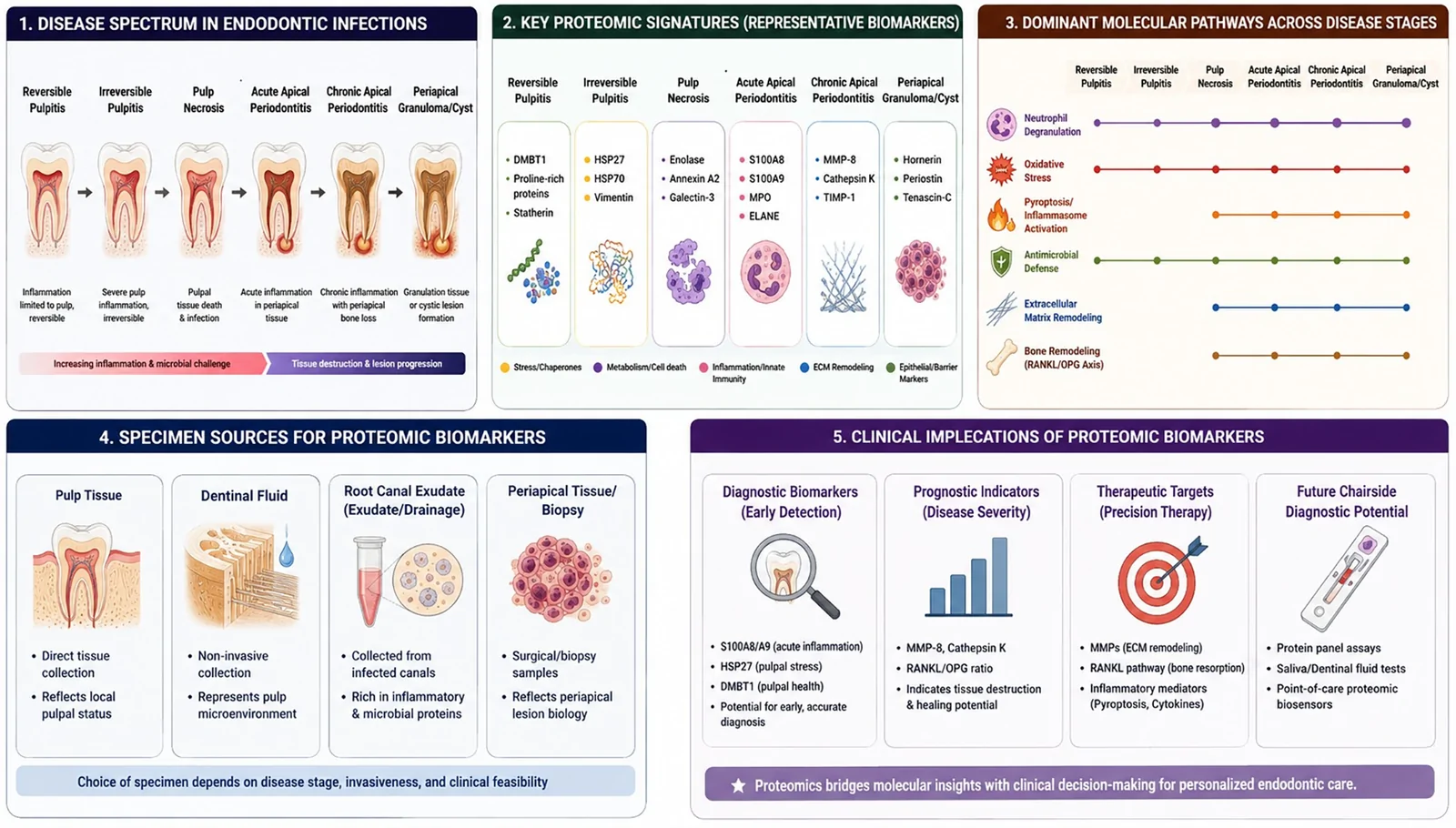
Schematic summary figure illustrating: (1) disease spectrum from reversible pulpitis to periapical cyst along the top axis; (2) key proteomic signatures per disease stage (hornerin, S100-A8/A9, HSP27, SERPINB1, MPO, pyroptosis markers, Gremlin-1); (3) enriched molecular pathways (neutrophil degranulation, oxidative stress, pyroptosis, bone remodeling); (4) specimen source for each biomarker; (5) clinical translation implications at the bottom axis (diagnostic, prognostic, therapeutic targets, chairside applicability).

Figure 2— Schematic summary figure illustrating: (1) disease spectrum from reversible pulpitis to periapical cyst along the top axis; (2) key proteomic signatures per disease stage (hornerin, S100-A8/A9, HSP27, SERPINB1, MPO, pyroptosis markers, Gremlin-1); (3) enriched molecular pathways (neutrophil degranulation, oxidative stress, pyroptosis, bone remodeling); (4) specimen source for each biomarker; (5) clinical translation implications at the bottom axis (diagnostic, prognostic, therapeutic targets, chairside applicability).

### 4.9 Conclusions

This systematic review provides the first comprehensive synthesis of proteomic profiling studies in pulpal and periapical diseases, revealing distinct molecular signatures that characterize disease stages, clinical presentations, and treatment outcomes. Eleven studies encompassing 577 participants identified over 1,800 human proteins and 720 microbial proteins across diverse biological specimens including dental pulp tissue, dentinal fluid, root canal samples, and periapical tissues. Key biomarkers including S100 family proteins, heat shock proteins, peroxiredoxins, myeloperoxidase, serpins, hornerin, and hemoglobin subunits emerged as consistent features of endodontic pathology, reflecting activation of neutrophil degranulation, oxidative stress response, inflammation, pyroptosis, and bone remodeling pathways. Proteomic signatures distinguished reversible from irreversible pulpitis, symptomatic from asymptomatic apical periodontitis, and stable from active periapical lesions, demonstrating potential for molecular diagnostics and prognostics. Metaproteomic analyses revealed complex host-pathogen interactions involving virulence factors, antibiotic resistance proteins, and immune evasion mechanisms that explain treatment failures. Despite significant advances, current studies are limited by small sample sizes, heterogeneous methodologies, lack of independent validation, and absence of longitudinal outcome correlations. Future research should focus on large-scale validation studies with standardized protocols, integration of multi-omics approaches, development of targeted assays for clinical translation, and prospective trials demonstrating clinical utility. By bridging the gap between molecular discovery and clinical application, proteomic profiling holds promise to transform endodontic practice through precision diagnostics, personalized treatment planning, and novel therapeutic strategies that improve patient outcomes.

## Data Availability

The original contributions presented in the study are included in the article/Supplementary Material, further inquiries can be directed to the corresponding author.
